# Current research trends and hotspots of boron neutron capture therapy: a bibliometric and visualization analysis

**DOI:** 10.3389/fonc.2024.1507157

**Published:** 2024-12-12

**Authors:** Yuyang Cong, Muyasha Abulimiti, Yoshitaka Matsumoto, Jing Jin

**Affiliations:** ^1^ Department of Radiation Oncology, National Cancer Center/National Clinical Research Center for Cancer/Cancer Hospital & Shenzhen Hospital, Chinese Academy of Medical Sciences and Peking Union Medical College, Shenzhen, China; ^2^ Department of Radiation Oncology, Faculty of Medicine, University of Tsukuba, Tsukuba, Ibaraki, Japan; ^3^ Department of Radiation Oncology, National Cancer Center/National Clinical Research Center for Cancer/Cancer Hospital, Chinese Academy of Medical Sciences and Peking Union Medical College, Beijing, China

**Keywords:** boron neutron capture therapy, bibliometric analysis, research trends, VOSviewer, CiteSpace

## Abstract

**Purpose:**

This study aimed to describe the trends, current hotspots, and future directions in boron neutron capture therapy (BNCT) through a bibliometric analysis.

**Methods:**

Articles related to BNCT published before 2023-12-31 were retrieved from the Web of Science Core Collection database. VOSviewer, R, and CiteSpace were used for bibliometric analysis and visualization.

**Results:**

A total of 3347 related publications from 1975 to 2023 were retrieved. Since a burst of published documents in 1992, the past three decades have witnessed continuous investigations into BNCT-related studies. Japan was the most productive country (794, 23.72%), followed by the USA (792, 23.66%), while the latter had the most citations. Kyoto University was the most influential institution. Ono K was the most prolific author, and *Applied Radiation and Isotopes* was the most popular journal. Ono K was the author that had the most total citations, followed by Barth RF. “Carborane”, “boronophenylalanine”, “glioblastoma”, “sodium borocaptate”, “cancer” and “drug delivery” were the most frequent keywords. The article “Dendrimers and dendritic polymers in drug delivery” had the most citations, whereas “Boron delivery agents for neutron capture therapy of cancer” had the highest outbreak value.

**Conclusion:**

Over the past three decades, research on BNCT has expanded significantly, with the development of novel boron carriers with improved medicinal characteristics being the most extensively investigated area. Future research will likely focus on the validation and modification of current BNCT treatment modalities using conventional boron agents in brain tumors, accelerator-based neutron sources and the application of BNCT in more clinical scenarios.

## 1 Introduction

Cancer remains one of the most devastating diseases affecting humans. In 2022, it was responsible for approximately one in six deaths globally, with an estimated 9.7 million fatalities (1). A major hinderance to the definitive cure of cancer is the therapeutic ratio, which has yet to increase owing to the inherent limitations of current treatment modalities. With respect to oncological surgery, the established principle of securing wide resection margins undoubtedly improved patient outcomes; however, a few residual tumor cells beyond the resection margins can lead to recurrence and metastasis (2). Systemic therapies can effectively eliminate disseminated malignant cells, but their side effects are common and sometimes lethal (3, 4). In the field of radiation therapy (RT), considerable effort has been devoted to the precise delivery of planned ionization radiation to the designed target volume and nowhere else (5, 6). Nonetheless, unintended doses to adjacent vulnerable organs at risk (OARs) limit its application to post-RT recurrences or tumors in critical locations.

**Figure 1:**
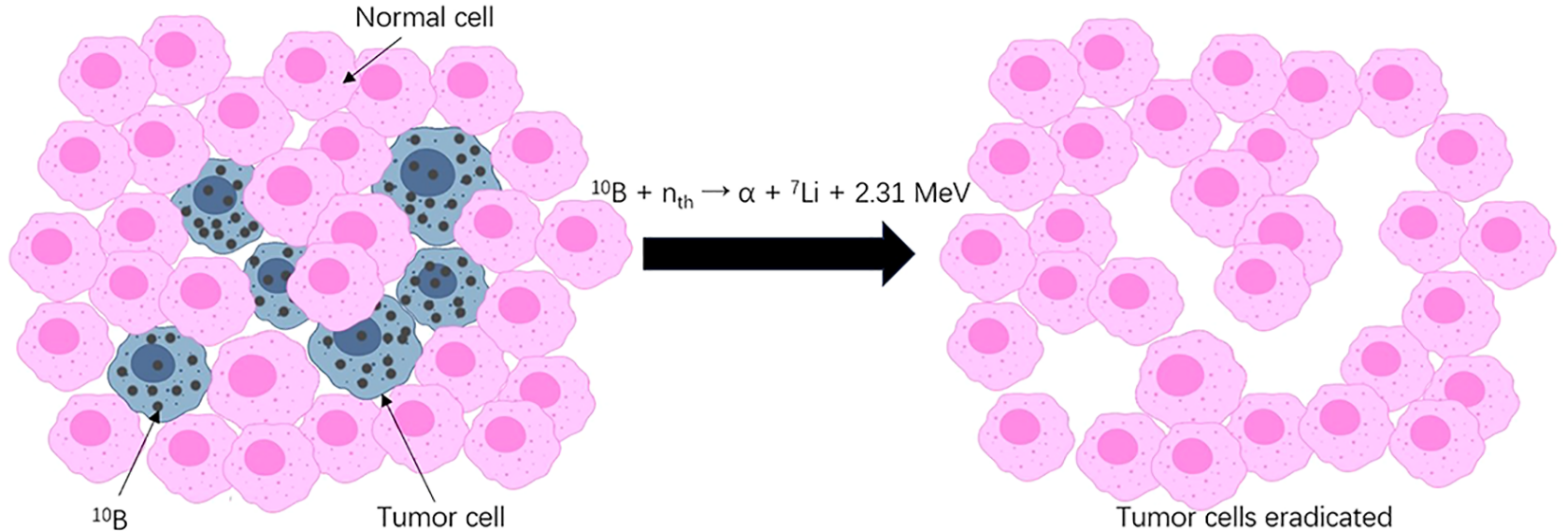
Ideal instance of BNCT.

Boron neutron capture therapy (BNCT), in contrast, is a unique two-step radiotherapy featuring unprecedented precision (**Figure 1**). In the first step, the nonradioactive isotope boron-10 (10B)-containing agent is administered and enriched in tumor cells. Second, with the selectively accumulated 10B, slow “thermal” neutrons radiated to the target volume induce neutron capture and decay reactions within a cell range (7). In this double-targeted manner, tumor cells, even those entangled with normal cells, receive a relatively high dose of irradiation and are thus susceptible to physical and biological destruction. Sparing nonmalignant components when killing tumors at a cellular resolution is a characteristic of BNCT that makes it a promising remedy for traditionally difficult-to-treat tumors.

Bibliometrics, first debuted in 1987, is a powerful tool for analyzing publication characteristics, canonical articles, and research trends in a specific field (8). By collecting most, if not all, related publications and utilizing statistical algorithms, bibliometric analysis could provide objective and comprehensive insights into the research area compared with conventional reviews that are dependent on the authors’ viewpoint. However, this useful tool has not been applied to BNCT research. Therefore, we conducted this study to fill this gap, aiming at describing the entire body of knowledge and helping researchers interested in BNCT grasp the whole picture of the past, the present and the possible future.

## 2 Methods

### 2.1 Data acquisition

**Figure 2:**
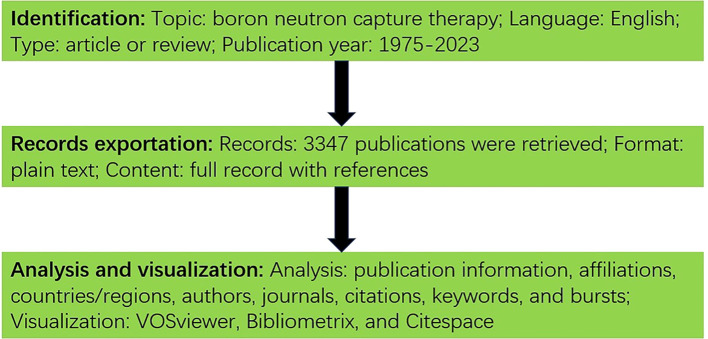
Research process of this study.

The following query line was used to retrieve publications from the Web of Science core collection (WoSCC): TS= (boron neutron capture therapy) AND LA= (English) AND DT= (Article OR Review). Related articles and reviews in English published before 2023-12-31 were collected. The search results were exported in plaintext format. Two authors independently conducted the literature screening, data retrieval and analysis to reduce bias in the results. The workflow is shown in **Figure 2**.

### 2.2 Bibliometrics and visualization analysis

VOSviewer 1.6.20), R (4.4.1), and CiteSpace (6.3. R1) were used in this study. VOSviewer was used to analyze the statistical characteristics of countries, institutions, journals, authors and keywords. CiteSpace was used to identify bursts of keywords and citations. Bibliometrix, a built-in R package, was used for data visualization (9).

## 3 Results

### 3.1 Publication characteristics

**Figure 3:**
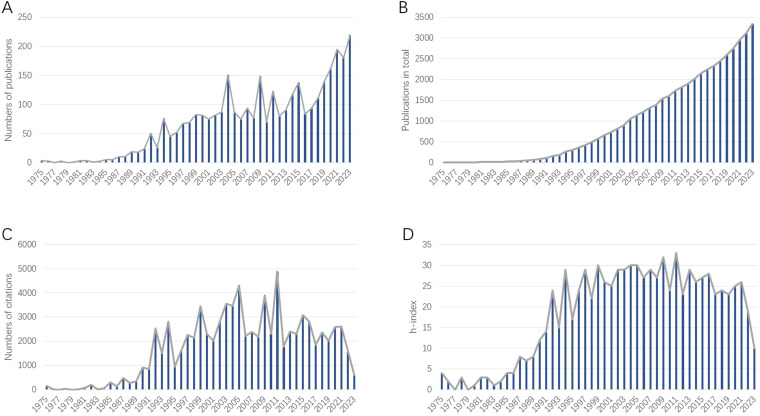
**(A)** Annual publications related to BNCT research. **(B)** Cumulative publications related to BNCT research. **(C)** Annual citations of the publications related to BNCT research. **(D)** Annual h-index of BNCT publications.

In total, 3347 publications related to BNCT were retrieved from the WoSCC (see **Table 1**). The annual publication numbers from 1975 to 2023 are shown in **Figure 3A**. We observed a surge of publications in 1992, followed by a 2.73% average annual growth rate, indicating that the attention attracted to BNCT research steadily increased. The year that had the most publications was 2023 (220, 6.57%). The total number of published articles steadily and rapidly increased from 1975 to 2023 (**Figure 3B**). From 1992 to 2021, the annual total number of citations was greater (**Figure 3C**), and the annual h index was 15 or greater, while it declined afterward (**Figure 3D**).

**Table 1:** Main information of the BNCT papers included in this study.

Description	Results
INFORMATION	
Timespan	1975-2023
Sources	688
Documents	3347
Annual growth rate	2.73%
Average citation per document	24.95
References	61368
DOCUMENT CONTENTS	
Keywords Plus (ID)	4290
Author’s Keywords	5181
AUTHORS	
Authors	10318
Authors of single-authored documents	105
AUTHOR’S COLLABORATION	
Single authored documents	141
Coauthors per document	6.42
International coauthorships	22.47%
DOCUMENT TYPES	
Article	2614
Article; book chapter	34
Article; early access	9
Article; proceeding papers	446
Article; retracted publication	1
Review	240
Review; book chapter	2

### 3.2 Countries/regions and institutions

**Figure 4:**
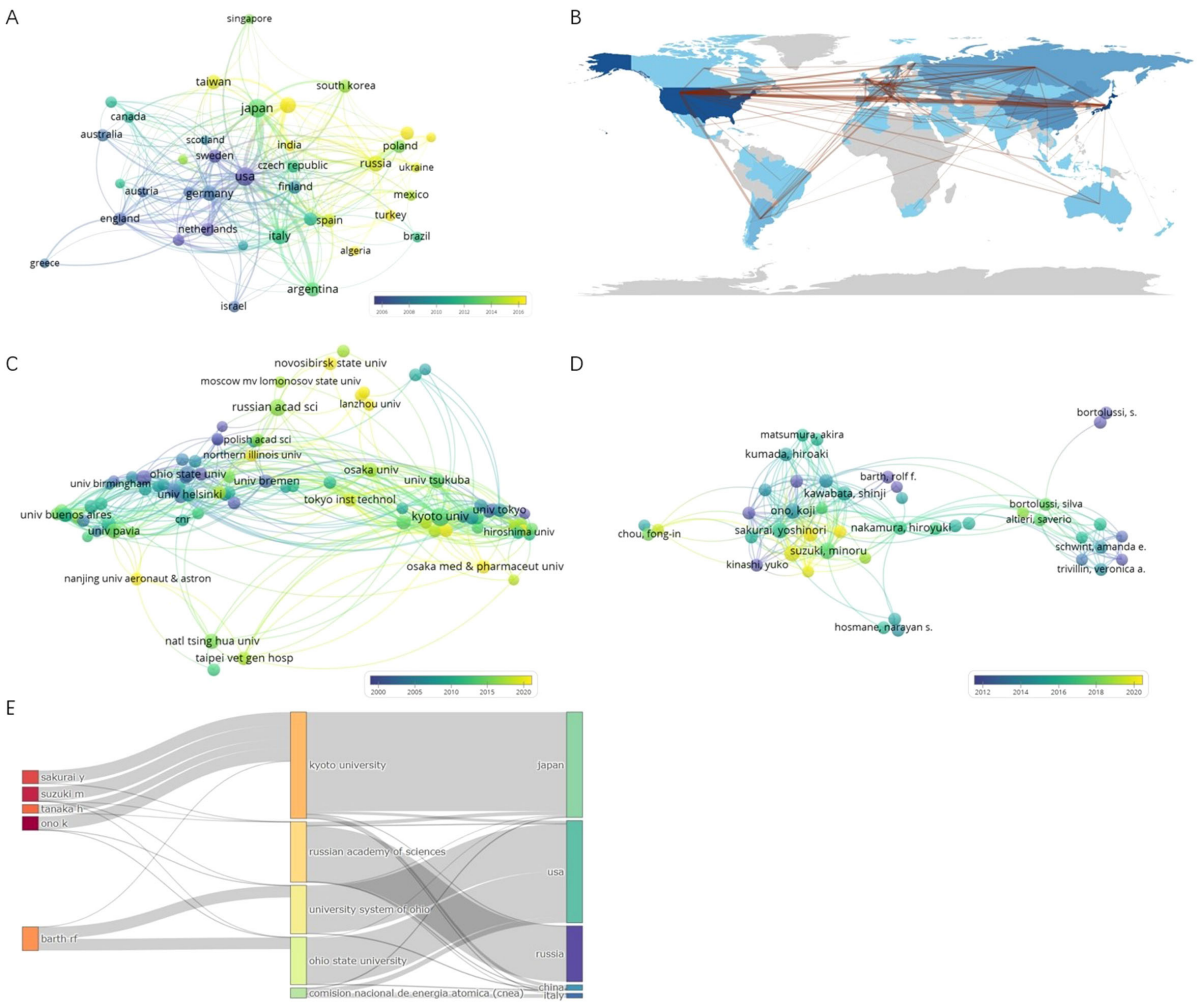
**(A)** Coauthorship overlay of countries/regions. **(B)** Geographic distribution of BNCT publications and cooperation strengths. **(C)** Coauthorship overlay of institutions. **(D)** Coauthorship overlay of authors. **(E)** Three fields plot of authors, affiliations and countries/regions.

A total of 79 counties/regions have contributed to research related to BNCT. The 38 countries/regions with more than 10 publications are shown in **Figure 4A**. The overlay network analysis of coauthorship reflects the number of publications by circle size and the average commencement year of study by color. In this two-dimensional diagram, strongly related nodes are located close to each other while weakly related nodes are located far away from each other. The link strength (LS) between nodes reflects the cooperation between countries, and the total link strength (TLS) is the sum of the LSs of a certain node. The USA had the strongest global cooperation (TLS=383) and cooperated the most with Argentina (LS=49). The top ten countries/regions with the highest number of publications and total citations are presented in **Table 2**. Japan had the most publications (794, 23.72%), followed by the USA (792, 23.66%) and Russia (274, 8.19%). Notably, the USA had the highest number of citations, followed by Japan. **Figure 4B** shows the geographic distribution of BNCT publications and cooperation strengths.

**Table 2:** The top 10 most productive countries/regions regarding BNCT from 1975 to 2023.

Rank	Country/Region	Publication	Fraction	Total citations
1	Japan	794	23.72%	17094
2	USA	792	23.66%	31414
3	Russia	274	8.19%	4769
4	Italy	265	7.91%	5466
5	Mainland China	251	7.50%	5287
6	Germany	209	6.24%	6082
7	Argentina	149	4.45%	2215
8	Taiwan	116	3.47%	1385
9	England	105	3.14%	3164
10	Sweden	101	3.02%	2989

In total, 2234 organizations have participated in BNCT research. **Figure 4C** shows 148 institutions with more than 10 publications. Kyoto University had the most cooperative relationships (TLS=624). The top 10 most productive institutions are listed in **Table 3**. Kyoto University was the most productive (355, 10.61%), followed by the Russian Academy of Sciences (186, 5.56%) and Ohio State University (138, 4.12%). Ohio State University had the highest number of total citations (7873). **Figure 4C** shows that in the past 30 years, the Massachusetts Institute of Technology (MIT), Brookhaven National Laboratory, and Ohio State University conducted BNCT research earlier, whereas Kyoto University, the Russian Academy of Sciences and the University of Tsukuba commenced later.

**Table 3:** The top 10 most productive institutions regarding BNCT research from 1975 to 2023.

Rank	Institution	Publication	Fraction	Total citations	TLS
1	Kyoto University	355	10.61%	7482	624
2	Russian academy of sciences	186	5.56%	3211	137
3	Ohio State University	138	4.12%	7873	109
4	University of Tsukuba	106	3.17%	1878	164
5	National Tsing Hua University	98	2.93%	1175	126
6	Brookhaven National Laboratory	96	2.87%	4636	106
7	Università di Pavia	90	2.69%	1519	190
8	Massachusetts Institute of Technology	74	2.21%	3460	128
9	Osaka Medical and Pharmaceutical University	73	2.18%	2519	176
10	Osaka University	70	2.09%	1823	118

### 3.3 Authors

A total of 8,574 authors have contributed to publications on BNCT. The top 10 authors with the most publications are shown in **Table 4**. Ono K had the highest efficiency (191, 5.71%), followed by Suzuki M (187, 5.59%) and Sakurai Y (163, 4.87%). Ono K had the highest total number of citations, whereas Barth RF had the highest h-index. **Figure 4D** shows a map of overlay network analysis among 236 researchers of 10 articles or more. Ono K cooperated the most with the others (TLS=1314). **Figure 4E** shows the correlations among the five most prolific authors, institutions, and countries that have contributed to BNCT research from 1975 to 2023 via a Sankey diagram (10).

**Table 4:** The top 10 most productive authors in BNCT research from 1975 to 2023.

Rank	Author	Publication	Fraction	Total citations	h-index	Start year
1	Ono, K	191	5.71%	5295	41	1996
2	Suzuki, M	187	5.59%	4047	33	1997
3	Sakurai, Y	163	4.87%	3235	30	1997
4	Tanaka, H	113	3.38%	1370	32	2009
5	Masunaga, S	87	2.60%	1888	25	1993
6	Coderre, J	78	2.33%	4497	37	1987
7	Nakamura, H	69	2.06%	1733	25	1993
8	Kawabata, S	68	2.03%	2377	27	2003
9	Kumada, H	68	2.03%	1414	22	2002
10	Barth, R	65	1.94%	4926	45	1984

### 3.4 Journals

**Figure 5:**
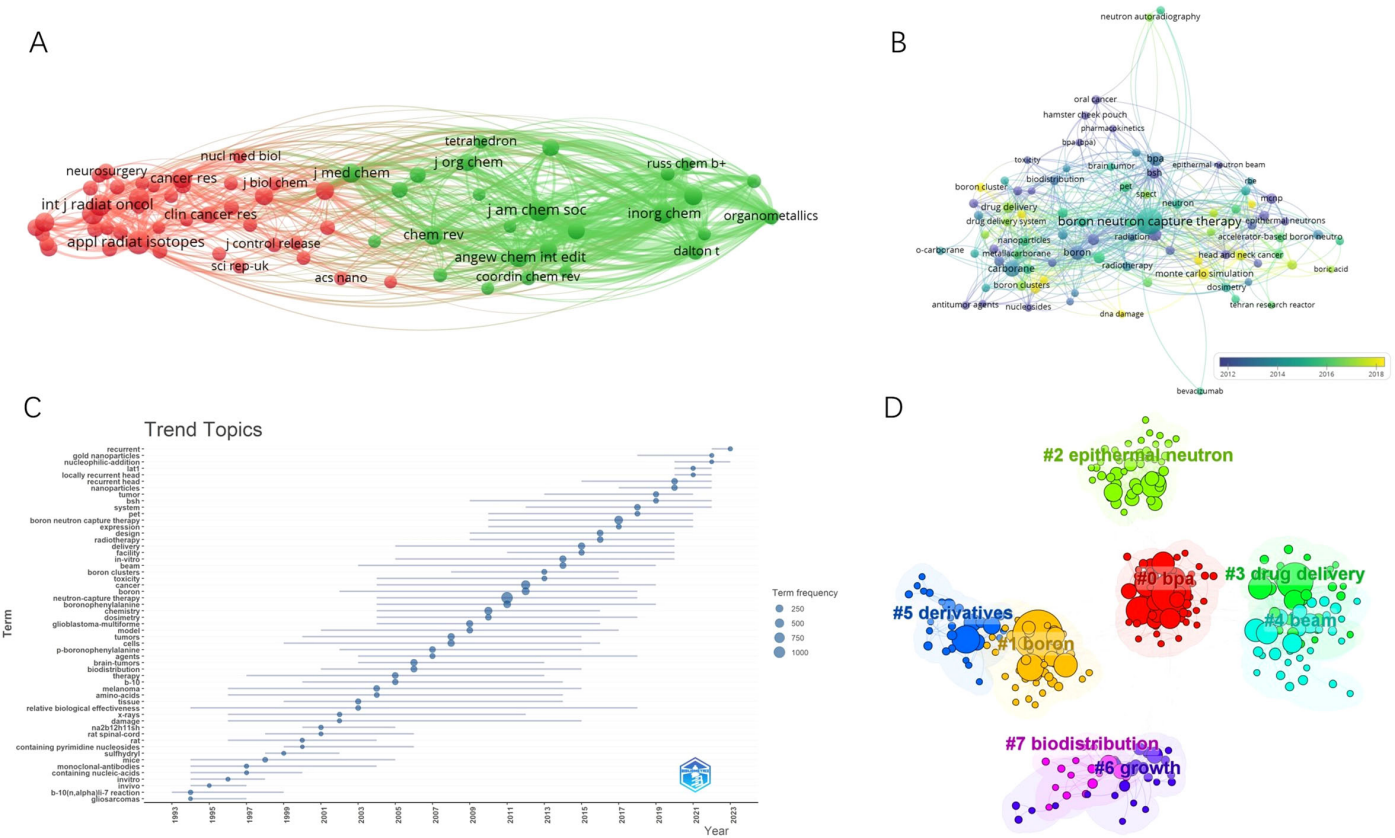
**(A)** Cocitation network map of journals. **(B)** Keyword co-occurrence network diagram of BNCT. **(C)** The trending topics. **(D)** Eight representative clusters of keyword clusters from 2019 to 2023.

A total of 687 journals have published articles related to BNCT. The top 10 journals in terms of BNCT publications are listed in **Table 5**, along with their publication counts, total citations, average citations, impact factor (IF) and Journal Citation Reports (JCR) quantile rankings. *Applied Radiation and Isotopes* had the most publications, followed by *Medical Physics and Nuclear Instruments and Methods in Physics Research Section A*. *Applied Radiation and Isotopes* had the highest total number of citations (5116), whereas the *Journal of Neuro-Oncology* had the highest average number of citations (50.49). The *International Journal of Radiation Oncology Biology Physics* had the highest IF (6.4), followed by *Physics in Medicine & Biology* (3.3) and the *Journal of Neuro-Oncology* (3.2). Most journals were classified as Q2 and above (90%) by JCR ranking quantiles. Journal directions included nuclear medicine, radiotherapy, oncology, and clinical neurology. A map of the cocitation network analysis is shown in **Figure 5A**. The top 3 cocited journals were *Applied Radiation and Isotopes* (4627), the *Journal of the American Chemical Society* (3286), and the *International Journal of Radiation Oncology, Biology, Physics* (3086).

**Table 5:** The top 10 most productive journals regarding BNCT research from 1992 to 2023.

Rank	Journal	Publications	Total citations	Average citations	IF	JCR
1	Applied Radiation and Isotopes	363	5116	14.09	1.6	Q2
2	Medical Physics	101	1954	19.35	3.2	Q1
3	Nuclear Instruments and Methods in Physics Research Section A	82	879	10.72	1.5	Q2
4	Radiation Research	73	2530	34.66	2.5	Q2
5	Radiation Protection Dosimetry	72	539	7.49	0.8	Q4
6	International Journal of Radiation Oncology Biology Physics	66	2756	41.76	6.4	Q1
7	Physics in Medicine & Biology	59	1494	25.32	3.3	Q1
8	Journal of Neuro-Oncology	55	2777	50.49	3.2	Q2
9	Nuclear Instruments and Methods in Physics Research Section B	46	741	16.11	1.2	Q1
10	Journal of Organometallic Chemistry	45	1150	25.56	2.1	Q2

### 3.5 Citation and cocitation analysis

Citation analysis is a valuable way to evaluate the most cited articles, and the number of citations can reflect the impact of an article in a particular field of research. The most cited articles by year in the realm of BNCT from 1975 to 2023 are listed in **Supplementary Table 1**. Among the 47 publications, 20 appeared in journals focused on organic and inorganic chemistry, 15 in journals dedicated to oncology and radiotherapy research, 7 in journals related to medicinal studies, and 5 in journals covering physics. Additionally, 30 of the 47 studies concentrated on the synthesis and properties of new boron-containing compounds, 10 on BNCT for cancer treatment, 5 on dosimetry, and 2 on the biological effects. In **Supplementary Table 2**, we summarize the 100 most cited articles in this field. The article “Dendrimers and Dendritic Polymers in Drug Delivery”, published in Drug Discovery Today in 2005, has received the highest citation count (1158).

### 3.6 Keywords

In total, 5181 keywords were proposed by the authors. A map of the overlay network analysis of keywords is shown in **Figure 5B**. The top 10 most common keywords were “BNCT”, “carborane”, “boronophenylalanine (BPA)”, “boron”, “glioblastoma (GBM)”, “sodium borocaptate (BSH)”, “cancer”, “drug delivery”, “neutron capture therapy”, and “Monte Carlo”. The terms marked in dark blue represent an average publication year of 2008 or earlier, whereas those in bright yellow represent 2016 or later. “BSH”, “GBM”, and “radiation” were previously the main topics. The keywords “Monte Carlo simulation”, “accelerator-based neutron source”, “cytotoxicity”, “drug delivery”, “head and neck cancer”, and “boron cluster” appeared relatively late. A similar trend is shown in **Figure 5C**.

To obtain the latest research hotspots of BNCT research, we analyzed the clustering of keywords within 5 years. Eight major clusters are shown in **Figure 5D** with their sizes and silhouette values in **Supplementary Table 3**. On the basis of clustering vocabulary analysis, the latest BNCT studies have focused on BPA administration, sources of neutrons, synthesis of novel boron-containing agents, their biodistribution, and the influence on tumor growth in animal models.

### 3.7 Citation bursts

**Figure 6:**
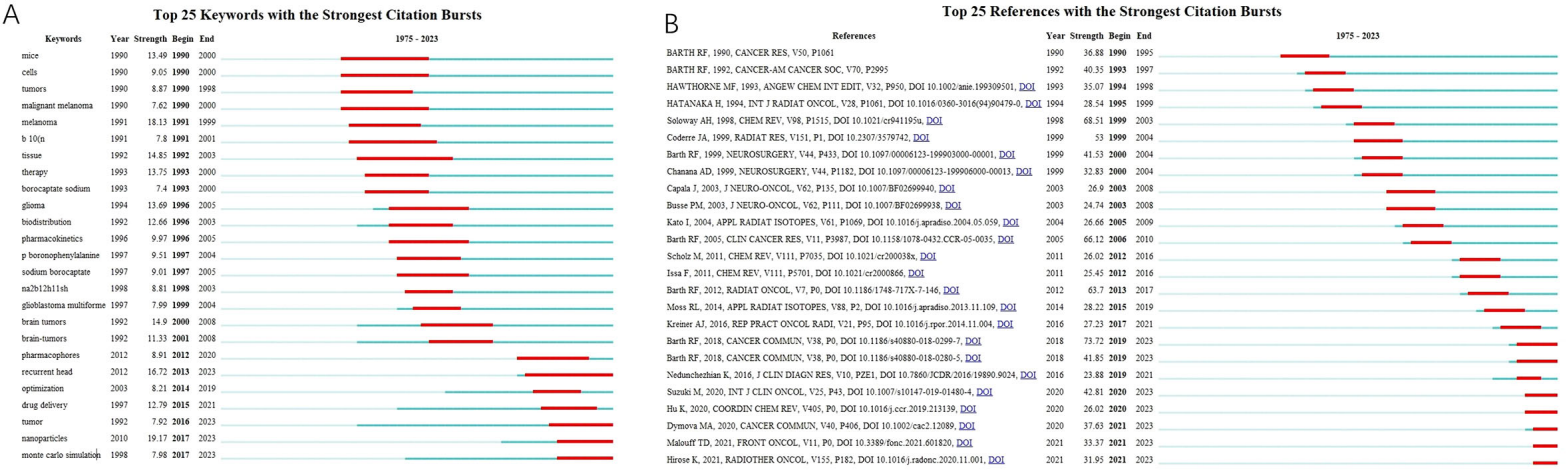
**(A)** The top 25 keywords and **(B)** references with the strongest citation bursts.

The top 25 keywords with citation bursts are shown in **Figure 6A**. The red lines reflect the burst duration. The keywords “mouse” (1990-2000) and “malignant melanoma” (1990-2000) drew considerable attention at the end of the 20^th^ century, whereas “glioma” (1996-2005), “glioblastoma multiforme” (1999-2004), “brain tumors” (2000-2008), and “BSH synonyms” (1993-2005) suggested a surge of publications related to the clinical outcomes of BNCT in the treatment of brain tumors using BSH as a boron carrier. In the second decade of the 21^st^ century, “recurrent head” (2013-2023), “drug delivery” (2015-2021), “nanoparticles” (2017-2023), and “Monte Carlo simulation” (2017-2023) have attracted extensive attention. The top 25 references with burst citations are shown in **Figure 6B**. The article “Boron delivery agents for neutron capture therapy of cancer” published by RF Barth et al. in Cancer Communications had the strongest outbreak, and the burst is still ongoing. The review “The Chemistry of Neutron Capture Therapy” by Soloway AH et al. had the second highest burst strength.

### 3.8 Trends of contribution

**Figure 7:**
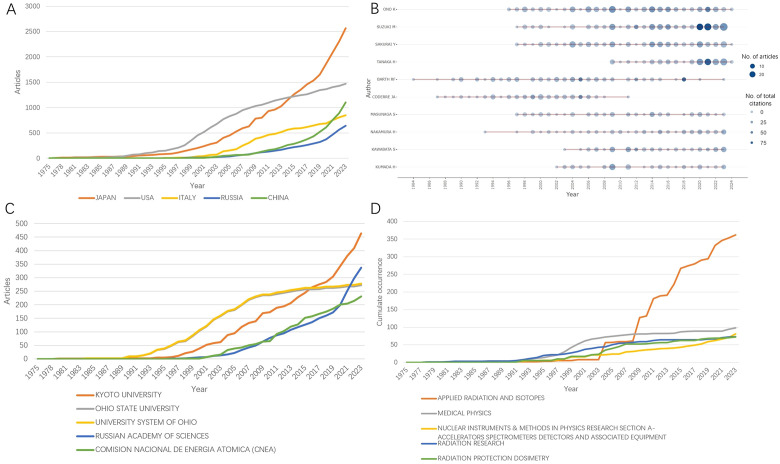
The production of top 5 **(A)** countries, **(B)** authors, **(C)** affiliations, and **(D)** journals over time.

The production of top 5 countries, authors, affiliations, and journals over time is shown in **Figure 7**, respectively. As demonstrated in **Figure 7A**, the cumulate contribution of Japan surpassed that of USA in 2015, while China’s production exceeded Italy’s in 2021. In the last five years, the most prolific authors are Suzuki M, Tanaka H, and Sakurai Y from Japan, while Kyoto University and Russian academy of sciences are the most active institutions worldwide (**Figures 7B, C**). Top five journals of BNCT research are shown in **Figure 7D**. From late 2000s, the journal *Applied Radiation and Isotopes* has been the leading source of BNCT related articles.

## 4 Discussion

### 4.1 Trends in the development of BNCT-related research

To date, this is the first study to offer a comprehensive bibliometric analysis of BNCT-related publications in the WOSCC from 1975 to 2023. The findings show a consistent increase in scientific output in this field. The peak number of citations and the highest H-index occurred in 2011. Japan, China, and Russia are the top three most productive countries in the recent five years. The decrease in citations and the h-index since 2022 may be attributed to the proximity to the end of the collection period.

### 4.2 Contributions by countries, institutions, authors, and journals

We conducted this bibliometric analysis in the field of BNCT to objectively identify the most influential countries, institutions, authors, and journals. Japan has emerged as a major contributor with the highest number of publications, whereas the USA has led in citations and international collaboration. Notably, three of the top ten institutions and eight of the top ten most productive authors are Japanese. Kyoto University was the most influential institution, and Ono K was the most prolific author over the past 30 years. The journal *Applied Radiation and Isotopes* had the most publications and total citations. Additionally, our results indicated that the *Journal of Neuro-Oncology* had the highest number of citations per publication in this field, suggesting that clinical applications of BNCT for GBMs continue to attract the attention of physicians and researchers.

### 4.3 Research hotspots and frontiers

To find research hotspots and leading edges in the field of BNCT, we conducted an analysis of citations, cocitations, and keywords. To our knowledge, this study is the only bibliometric analysis of this topic, with several meaningful conclusions. First, the synthesis of novel boron-containing agents is a constant hotspot, varying from boronic acid derivatives lacking selectivity to boron clusters such as carboranes and then to modern boron carriers with targeting capacity. Antibody conjugates and nanoparticles are becoming more attractive to researchers. Our keyword analysis results indicate that researchers have paid increasing attention to “accelerator-based neutron source”, “cytotoxicity”, “drug delivery”, “head and neck cancer”, and “boron cluster”, with the aim of improving the neuron source, boron delivery and number of types of cancer to treat with BNCT. In contrast, previous studies focused more on BSH and GBMs. Third, the burst analysis of keywords and citations revealed “brain tumors”, “nanoparticles”, and “Monte Carlo simulation”, suggesting that they are milestones in the development of BNCT research. Finally, with the addition of cutting-edge publications, we sense prelusive therapeutic advancements using novel boron agents, with applications unrestricted to small animals in the near future.

#### 4.3.1 Development of neutron sources

Ideal neutrons for BNCT should be radiated at high flux rates, designated energy levels and targeted volumes without unwanted heavy particles. Although the characteristics and purity of the neutrons were subpar with respect to modern standards, experimental nuclear reactors provided the first thermal neutrons that contributed to the proof of concept of BNCT. One decade after World War II, Farr LE and Sweet WH used the Massachusetts Institute of Technology nuclear reactor (MITR) as neutron sources (11). In the 1960s, Hatanaka initiated clinical trials on brain malignancies via the Hitachi Training Reactor and the Musashi Institute of Technology reactor (12). As mentioned above, the results of these trials were far from fruitful. The slow neutrons had a critical disadvantage. Owing to their low energy level, thermal neutrons can only pass through an average of 2 cm in the tissue before they lose the capacity to induce a nuclear reaction. This resulted in intracranial tumor patients often needing debulking resection and the surgical cavity being left open when receiving the external beam, which might have led to brain necrosis by increasing the boron concentration at exposed incisions (13). In addition, the short path length of thermal neutrons has limited the noninvasive application of BNCT to melanomas and other superficial neoplasms.

Reactors remained the mainstream neutron source until the early 21^st^ century as the physical characterization of their neutrons improved. The epithermal neutrons from MITR II and Brookhaven Medical Research Reactor (BMRR) with modified beam parameters came into clinical use for melanoma and GBM patients in the 1990s (14, 15). The relatively high-energy epithermal neutrons (0.5 eV < E_n_ < 10 keV) from reactors had better penetration of approximately 6 cm beneath the tissue surface, comparing to 2 cm for thermal (E_n_ < 0.5 eV) neutrons, which significantly increased the body volume in reach and made craniotomy unnecessary (14, 16). However, several limitations of reactor-based BNCT exist. First, the rareness of a nuclear reactor is a major impediment to BNCT research. The technical requirements and financial burdens of building and maintaining a nuclear reactor dwarf the benefits of this promising solution as definitive precision medicine. In addition, the reactors need to be shared with basic physics researchers and shut down for maintenance for one third of a whole year (17). Finally, nuclear accidents are always a concern.

Alternative neutron sources have been under development since the 1980s (18). Accelerators at that time for physical research had a neutron density far less than the requirement of BNCT, which is more than 1×10^11^ to 10^12^ (n/cm^2^·s) (19). In recent years, accelerator-based (AB) systems have provided comparable intensities of epithermal neutrons with the whole system in a compact form suitable for in-hospital installation (20). In short, protons are accelerated by cyclotrons (21) or linear accelerators (22) to hit the target metal materials, such as lithium (23) or beryllium (24). Nuclear reactions, e.g., ^7^Li(p, n)^7^Be or ^9^Be(p, n)^9^B, occur, and then, epithermal neutrons are emitted from the target. By 2023, AB-BNCT has become standard clinical therapy covered by medical insurance in 5 hospitals in Japan (25). Currently, several clinical studies in China (26) and Korea (27) in which several manufacturers use accelerator-based neutron sources (ABNSs) are in progress. The clinical application of ABNS in the new era warrants further investigation.

#### 4.3.2 Development of boron delivery agents

The development of boron carriers has spanned over 70 years and remains critical to the success of BNCT. The general requirements for boron agents include low systemic toxicity, high selectivity, and durable persistence in tumors during BNCT (28). The challenge lies in achieving at least 20 μg/g ^10^B in tumors for effective radiation, while less than one-third of the boron is present in blood or normal tissue. Advances in synthetic techniques and biological targeting have led to the emergence of several promising boron delivery strategies.

The first boron carrier used in early BNCT studies was borax. In Farr’s procedure, sodium tetraborate was infused intravenously, followed by 30 min of irradiation of given neutron counts at the skin surface, and a few derivatives of it were used in subsequent trials (11, 29). These first-generation compounds have poor selectivity, a low tumor tissue ratio, short persistence in brain tumors and high systemic toxicity (29). BSH (30) and BPA (31) are second-generation boron agents that are more selective and have been widely used in clinical trials. BSH is a polyhedral borane anion commonly used in Japanese clinical trials. The EORTC 11961 study revealed that the tumor-to-brain ratio of BSH was greater than 40 in GBM patients (32). BPA is a hydrophobic dihydroxyboryl derivative of phenylalanine that can be actively transported into cells via the L-amino acid transport system and is upregulated in most tumors (33). The safety of intravenous administration of BSH and BPA has been clinically tested in Japan, the USA, Europe, and Argentina (34), although neither meets all of the requirements. The main limitations of BSH are its lack of receptor-mediated tumor selectivity, low tumor-to-blood ratio, and significant side effects during treatment (35). While some researchers have addressed this by conjugating BSH with specific ligands (36, 37), its high cost remains a barrier to its use as an ideal boron carrier. The low boron content of BPA requires high doses, increasing treatment costs and straining liver and kidney metabolism. Its poor solubility and instability lead to a reduced boron concentration at the tumor site (38), whereas its short retention time, possibly influenced by LAT1’s anti-transport mechanism, further limits its effectiveness.

The next generation of boron compounds features the conjugation of boron agents to tumor-targeting molecules to achieve the selective accumulation of boron in tumors. The targeting relies on the binding of linked ligands to corresponding receptors that are overexpressed by tumor cells. These novel boron agents generally consist of a stable boron cluster or moiety of high boron concentration and tumor-targeting molecules linked together. Amino acids, peptides, carbohydrates, nucleosides, antibodies, and nanoparticles as targeting or carrier moieties have attracted considerable attention.

**Low-molecular-weight agents as targeting moieties.** In addition to BPA, boronated amino acids, along with their derivatives, have been widely investigated. These include natural amino acids and unnatural cyclic amino acids (39, 40). However, none of these compounds possess better *in vitro* characteristics than BPA derivatives do (35). Peptide ligands have been demonstrated through the functionalization of carriers with high affinity and selectivity (41). Michiue et al. reported that when connected to arginine repeats, BSH has increased intracellular accumulation in both the cytoplasm and nucleus (42). *In vivo* positron emission tomography (PET) confirmed the selective retention of BSH-3R in tumor tissue. Nagasawa et al. developed and evaluated a novel boron carrier by conjugating BSH to cell-membrane penetrating peptides (CPPs) (43). Compared with BSH, the CPP-conjugated form presented a greater intracellular concentration and better eradication of T98G cells when exposed to neutrons. Barth’s group proposed a strategy to target vascular endothelial growth factor receptor (VEGFR) and epidermal growth factor receptor (EGFR), which are often overexpressed in tumors (44, 45). However, indirect tumoricidal activity via ischemic necrosis and the expression of EGFR on normal glial cells have limited their success.

Cancer cells have an increased rate of glucose uptake and glycolysis, known as the Warburg effect (46), which renders carbohydrates an ideal ligand for boron conjugates. Aoki et al. synthesized highly hydrophilic 2-borylsugars that were actively transported into cancer cells by glucose transporter 1 (GLUT1). Then, 2-borylsugar is phosphorylated and stored intracellularly, as confirmed by *in vitro* assays (47). Ekholm et al. developed 6-O-carboranylmethyl glycoconjugates with high affinities for GLUT1 (48). Compared with second-generation boron agents, this compound has a 40-fold greater boron delivery capacity. Tsurubuchi et al. reported the synthesis of an α-D-mannopyranoside derivative named MMT1242 with prolonged intracellular retention compared with that of BPA (49). Notably, both mannose receptors (MRs) and GLUT1 mediate the uptake of MMT1242. Given that MRs are upregulated on the cell membranes of tumors and correlate with tumorigenesis (50), mannose conjugates hold potential for improving the therapeutic effects of BNCT. In summary, despite their high water solubility and rapid clearance from metabolically active tumor cells, leading to poor intracellular boron retention, these studies underscore the potential of carbohydrate-based boron carriers. Their enhanced uptake, high tumor affinity, and growth inhibition offer valuable insights for developing new boron agents for BNCT.

Other small molecules, such as nucleosides, porphyrins, folic acid (FA), hyaluronic acid (HA), and COX-2 substrates, have also been widely investigated in recent years. Thymidine kinase 1 (TK1) is highly expressed in the cytosol of proliferating tumor cells (51). To target TK1, Barth et al. synthesized a 3-carboranyl thymidine analog (3CTA), N5–2OH, with high tumor-to-brain and tumor-to-blood ratios (52). *In vivo* studies demonstrated a significantly prolonged mean survival time (MST) in rats bearing RG2 gliomas, whereas validation of N5–2OH in an F98 glioma model revealed minimal MST improvement (53). The underlying cause of this phenomenon needs further investigation. FA receptors are highly expressed in tumor cell lines and are correlated with a metastatic tendency (54, 55). In 2020, Nakagawa’s group synthesized several hydrophilic FA derivatives with IC_50_ values of less than 3 mM in the U87 MG glioma cell line (56). The HA receptor CD44 is upregulated in cancers of numerous origins (57). In 2008, Crescenzi’s group designed an HA-conjugated carborane, named HapACB, with high affinity for CD44 *in vitro* (58). COX-2 is highly expressed in oral squamous carcinoma cells (59). Boronated COX-2 inhibitors demonstrated great radiosensitization capacity in CAL27 cells by suppressing the PI3K/Akt and MAPK signaling pathways (60). The conjugation of boron-enriched moieties with well-validated targeting molecules might be a productive avenue in the future.

**High-molecular-weight agents as targeting moieties.** Monoclonal antibodies possess unparalleled targeting selectivity, and antibody-based therapies are achieving significant clinical success across various cancers (61). With respect to BNCT, the first antibody-assisted localization was achieved by conjugating benzenediazonium ions to antibodies against carcinoembryonic antigen (CEA) in 1984 (62). This conjugate contained 30 boron atoms per IgG molecule and demonstrated a high degree of selective accumulation in CEA-positive human colonic carcinomas grown in hamsters. Recently, EGFR and CD133 have been investigated as targets for antibody conjugates. Barth and colleagues developed dendrimers with high boron concentrations linked to cetuximab, a widely used monoclonal antibody that targets EGFR (63); or to L8A4, which targets EGFR_vIII_ (64); or to EGF (45) *per se*. These EGFR-binding boron agents demonstrated significant tumoricidal effects in a rat model bearing F98 gliomas transfected with the corresponding genes (63–66). Nakase’s group also targeted EGFR via the conjugation of dodecaborate to cetuximab through the Z33 peptide (67). EGFR-expressing cells internalize the conjugates via the macropinocytotic pathway, but further validation of BNCT therapeutics involving this compound is necessary. Sun’s group reported that with CD133 on SU2 glioma cells targeted by boron−antibody conjugates, the elongation of experimental mouse survival time was remarkable (68). To date, the key challenges of antibody-based boron delivery strategies include (1) coping with variable expression of the target across tumors (2); elucidating the mechanisms for entering cancerous cells; and (3) achieving uniform boron distribution or selective enrichment within critical compartments of the tumor.

As the burst analysis indicated, extensive investigations have recently been conducted on the use of nanocarriers for boron delivery. Nanoparticles are materials with specialized functions formed by atoms or molecules at the nanoscale. Recent progress in materials chemistry, biology, and related areas has rapidly advanced the use of nanoparticles in fields such as industry and medicine (69, 70). These nanoparticle-based systems have demonstrated numerous advantages in drug delivery, *e.g.*, high stability, water solubility, tumor accumulation, and low preparation requirements (71), due to the selective accumulation of nanoparticles in tumors, known as the enhanced permeability and retention (EPR) effect (72). Additionally, nanocarriers can easily deliver other therapeutic agents alongside boron compounds to tumors. As a result, nanostructures have emerged as a focus of BNCT research.

Polymers represent a prominent class of nanocarriers that have been extensively studied in recent years, such as dendrimers (73) and polymer micelles (74), which have the ability to extend drug retention in tumors and enhance the hydrophilicity of drugs. The boron-containing compounds are either conjugated to or encapsulated by the polymers. In 2012, Sumitani et al. reported significant suppression of colon-26 tumor (CT26) growth in mice treated with carboranes embedded by their poly(ethylene glycol)-block-poly(lactide) copolymer (PEG-b-PLA) (75). Five years later, Makino and coworkers reported that poly(L-lactide-co-glycolide) (PLLGA)-encapsulated carboranes maintained a tumor-to-blood ratio of boron concentration over 5 for more than 8 hours after administration (76). In 2019, Chen’s group developed a polymer-based tumor-targeting boron delivery system, named iRGD-PEG-PCCL-B (77). When wrapped by this polymer, BSH had six times higher uptake by A549 cells than its original form. In addition, iRGD-PEG-PCCL-B could simultaneously pack the BSH with doxorubicin, enabling highly targeted BNCT combined with chemotherapy for synergistic tumor suppression. A year later, Nomoto and colleagues prepared a novel polyvinyl alcohol-BPA complex that demonstrated improved uptake mediated by LAT1, prolonged cellular retention, and substantial CT26 growth suppression with high biocompatibility (78). In 2023, Dai et al. synthesized novel BPA-containing polydopamine (B-PDA) nanoparticles that had significant glioma ablation capacity in mice exposed to neutron radiation, with a tumor-to-brain ratio of 6.83 ± 1.75 (79). Overall, the use of polymers as boron carriers has provided substantial preclinical evidence, demonstrating their potential as promising candidates for further investigations.

Liposomes and inorganic boron-containing nanoparticles have undergone extensive investigation, as examined in two recent reviews (80, 81). These inorganic nanoparticles can be classified into boron nitride (82, 83) (BN) nanotubes (84–86), boron-doped carbon dots (87), magnetic nanomaterials (88, 89), gold nanoparticles (90, 91), and mesoporous silica nanoparticles (92, 93), although a more detailed review is beyond the scope of this study. Owing to their high biocompatibility, low systemic toxicity, improved physiochemical stability, controlled release and EPR effect, the majority of these multifunctional nanocomposites demonstrated improved selectivity for tumor cells and increased boron uptake in both *in vivo* and *in vitro* experiments through ligand conjugations, surface modifications or structural alterations, rendering these categories of nanocarriers promising blueprints for innovative boron delivery materials.

#### 4.3.3 Enhanced imaging and dosimetry

Traditional imaging techniques have offered limited insight into the distribution of boron compounds and the dose delivered to tissues. To analyze the boron concentration, various methods are available, including colorimetry, prompt γ-ray analysis (PGRA), inductively coupled plasma atomic emission spectrometry (ICP−AES), direct-current plasma atomic emission spectroscopy (DCP-AES), inductively coupled plasma−mass spectrometry (ICP-MS), quantitative neutron capture radiography (QNCR), electron energy loss spectroscopy (EELS), flow injection combined with ESI-MS/MS (FI/ESI-MS/MS), sputter-initiated resonance ionization microprobe (SIRIMP), laser atomization resonance ionization microprobe (LARIMP), secondary ion mass spectrometry (SIMS), single-photon emission computed tomography (SPECT), PET, nuclear magnetic resonance (NMR), and magnetic resonance imaging (MRI). All of the current boron drugs and their imaging techniques are presented in **Table 6**.

**Table 6:** List of current boron drugs and imaging techniques.

Boron Agent	Technique	Invasion	Years	Disadvantages	Used in Clinic
BSSB	11B-NMR	No	1988	Requires high-field NMR equipment; limited clinical use due to cost and availability.	No
BSH	DCP-AES (94)	Yes	1991	Less commonly used than ICP−AES; potential matrix effects.	No
BSH	PGRA (95)	Yes	1995	Limited to quantification; Unsatisfactory accuracy.	No
BSSB	SIRIMP (96)	Yes	1997	Limited to research applications; may not be widely accessible.	No
BSSB	LARIMP (96)	Yes	1997	High cost and complexity; requires precise calibration and sample preparation.	No
BSH	10B-NMR	No	2001	High cost and specialized equipment required; less common in clinical practice.	No
BSH	EELS (97)	Yes	2003	Requires specialized equipment; sample must be electron-transparent.	No
BSH, BPA	QNCR (98)	Yes	2005	Limited availability; primarily used in research settings.	No
BSH	FI/ESI-MS/MS (99)	Yes	2005	Complex and costly; requires expertise in mass spectrometry.	No
BSH, BPA	ICP−AES (100, 101)	Yes	2006	Requires sample preparation; may have interference from other elements.	No
cis-ABCPC, 10BPA, 11BSH	SIMS (102, 103)	Yes	2013 2002	High resolution but require extensive sample preparation; limited to research use.	No
BSH, BPA	laser-SIMS (104)	Yes	2008	Requires sophisticated equipment; not widely used in clinical settings.	No
BPA	nano-SIMS (105)	Yes	2019	Extremely high cost; complex data analysis and sample preparation.	No
BPA	ICP-MS (106)	Yes	2015	High cost; requires skilled operators and extensive sample preparation.	No
BPA	SPECT (107)	No	2000	Lower spatial resolution compared to other imaging techniques; radiation exposure.	Yes
10BPA	18F-BPA PET (108)	No	2018	High cost; requires radiopharmaceutical production and specialized equipment.	Yes
carborane	MRI (109)	No	2000	May require contrast agents; lower resolution for boron-specific imaging compared to other techniques.	Yes
BPA	MRI (110)	No	2005	Limited specificity for boron without specialized contrast agents; resolution may vary.	Yes

BSSB, Cs4B24H22S2; BSH, Borocaptate sodium; BPA, boronophenylalanine; cis-ABCPC, 1-amino-3-borono-cyclopentane carboxylic acid; NMR, nuclear magnetic resonance; DCP-AES, direct-current plasma atomic emission spectroscopy; PGRA, prompt γ-ray analysis; SIRIMP, sputter-initiated resonance ionization microprobe; LARIMP, laser atomization resonance ionization microprobe; EELS, electron energy loss spectroscopy; QNCR, quantitative neutron capture radiography; FI/ESI-MS/MS, the flow injection technique combined with ESI-MS/MS; ICP−MS, inductively coupled plasma−mass spectrometry; SPECT, single-photon emission computed tomography; PET, positron emission tomography; MRI, magnetic resonance imaging.

Currently, not all of these technologies are utilized in clinical settings. Many remain at the cellular level or in research stages, such as QNCR and SIRIMP, whereas newer methods such as nano-SIMS require additional development before clinical use. Presently, four primary methods are employed in clinical practice, all of which are based on modern imaging techniques: positron emission tomography (PET) and magnetic resonance imaging (MRI) provide enhanced visualization of the boron distribution and tumor response. Additionally, advanced dosimetry tools now enable precise measurement of radiation doses, ensuring accurate treatment planning.

#### 4.3.4 Clinical advances

Early clinical trials had mixed results due to technology and compound limitations. More recent clinical trials have demonstrated promising results, particularly with new boron compounds and improved neutron sources. Trials are exploring the effectiveness of BNCT for various cancers, including those resistant to conventional therapies. The evidence suggests better outcomes and fewer side effects in some cases.

**Glioblastoma multiforme:** The use of BNCT for treating brain gliomas can be traced back to 1951-1961 (111). During this period, Brookhaven National Laboratory conducted a clinical trial using 10B-enriched borax and recruited 28 patients with brain cancers, including GBM and other types of gliomas. However, these trials failed because the first generation of boron drugs could damage normal tissue and be cleared from tumor cells too quickly, resulting in insufficient boron concentrations in tumors. With the subsequent development of borocaptate sodium (BSH) and boronophenylalanine (BPA) compounds, a series of clinical findings emerged after the 1990s. Five trials conducted in Japan from 1998 to 2007 (112), 1998 to 2008 (113), 1999 to 2002 (114), and 2002 to 2007 (115) reported median survival times (MSTs) of 25.7 months, 19.5 months, 23.2 months, and 10.8 months, respectively. These trials involved 15, 23, 9, and 21 patients with brain cancer, and they used various medication regimens and radiation doses. This series of studies revealed that BNCT combined with X-ray and temozolomide could prolong the median survival time of patients with GBM. In the past 20 years, with the development of new boron drugs and advancements in dosimetry, seven clinical studies on GBM have been registered with the JRCT and NCT. A summary of the results is shown in **Table 7**. Two studies have reported preliminary results, namely, BNCT with temozolomide (TMZ) for GBM, with an MST of 15.6 months in patients with newly diagnosed GBM and 8.6 months in patients with recurrent GBM, with the remaining studies ongoing. Other studies are in the recruiting stage, and it is worthwhile to follow the results of these studies to better guide BNCT in treating GBM.

**Table 7:** List of Registered Clinical Studies of BNCT for the Treatment of Tumors in the Last 20 Years.

Number	Locations	Phase	Case	Study Status	Cancer Type	Interventions	Start Date	Completion Date	Results
NCT05737212 (116)	Korea	I/II	39	Recruiting	Brain and CNST Tumors	BNCT	2022/12/5	2024/12/1	Not reported.
NCT00974987 (117)	Japan	II	32	Completed	Brain and CNS Tumors	BNCT+XRT+TMZ	2009/9/1	2016/2/29	MST: 15.6 m
NCT01233492 (118)	United Kingdom	I	36	Terminated	Brain and CNS Tumors	BNCT+ mannitol	2007/10/1	2013/9/1	Not reported.
jRCT2032230554 (119)	Japan	I	18	Recruiting	Brain and CNS Tumors	BNCT+TMZ	2023/12/7	NA	Not reported.
jRCTs051180218 (120)	Japan	II	4	Recruiting	Brain and CNS Tumors	BNCT+TMZ	2019/3/27	2020/2/18	MST 8.5 m
NCT00062348 (121)	Germany	I	27	Completed	HNC	BNCT	2003/6/5	2012/1/20	Tumor/blood 1.2 ± 0.4. 1.4 ± 0.5 for skin
NCT01173172 (122)	Taiwan	I/II	17	Completed	HNC	BNCT	2010/7/1	2015/3/7	RR 71% 2yOS 47%
NCT02004795 (123)	Taiwan	I/II	28	Unknown	HNC	BNCT + IG-IMRT	2013/11/1	2018/11/1	Not reported.
NCT00114790 (124)	Finland	I/II	17	Completed	HNC	BNCT	2005/6/17	2013/5/6	RR 83%, MDOR 12.1 m
NCT05883007 (125)	Japan	I	30	Unknown	HNC	BNCT	2020/6/1	2024/5/31	Not reported.
jRCT2080224571 (126)	Japan	II	21	Completed	HNC	BNCT	2019/2/22	2021/1/25	3 m RR: 71.4%; 2y OS rate: 58% (rSCC12); 100% (r/la-nSCC13)
jRCTs051180160 (127)	Japan	II	7	Completed	HNC	BNCT	2019/3/25	2022/1/11	RR: 85.7%
jRCTs031180302 (128)	Japan	II	14	Completed	HNC	BNCT with PETCT	2019/3/15	2022/4/1	SUVmax = 4.5 +- 1.1/3.4 +- 0.8 T/N ratio = 3.6 +- 0.8/1.9 +- 0.6
UMIN000044118 (129)	Japan	II	120	Recruiting	HNC	BNCT	2021/5/10	NA	Not reported.
ChiCTR2200066473 (130)	China	II	6	Recruiting	HNC	BNCT	2022/09/19	2024/12/31	Not reported.
NCT02759536 (131)	China	I/II	30	Unknown	Melanoma	BNCT and IHNI-based BNCT	2013/7/1	NA	Not reported.
NCT00085059 (132)	Germany	II	4	Terminated	Melanoma	BNCT	2004/4/1	2012/7/18	None
NCT04293289 (133)	Japan	I	10	Completed	Melanoma Angiosarcoma	BNCT	2019/11/19	2022/12/31	Not reported.
NCT05601232 (134)	Japan	II	10	Recruiting	Angiosarcoma	BNCT	2022/11/1	2025/4/30	Not reported.

CNS, central nervous system MST, median survival time; TMZ, temozolomide; XRT, X-ray radiotherapy; IG-IMRT, image-guided intensity-modulated radiation therapy; RR, response rate; HNC, head and neck cancer; r/lHNC, recurrent/locally head and neck cancer; OS, overall survival; r/la-nSCC, recurrent/locally advanced nonsquamous cell carcinoma; a/rHNC, advanced/recurrent head and neck cancer; T/N ratio target/background ratio; IHNI, In-hospital Neutron Irradiator; MDOR, median duration of response; N, not available.

**Head and neck cancers:** BNCT has been used for various head and neck cancers, including squamous cell carcinoma (SCC). It provides a viable alternative to conventional therapies, especially for patients who have not responded well to traditional treatments or those with recurrent head and neck cancer (HNC). In the past 20 years, approximately 10 clinical trials have investigated BNCT for HNC. The earliest trial, EORTC 11011 (NCT00062348) (121), began in Essen, Germany, in 2003 and involved six patients with advanced SCC of the head and neck. These patients received an injection of either BPA or BSH, and the researchers assessed the distribution of boron-10 (B10) in both tumor and normal tissues. The mucosa and skin were identified as the most critical organs at risk.

Between 2010 and 2015, researchers in Taiwan conducted studies involving 17 patients who underwent BNCT with BPA at a dose of 400 mg/kg, delivering a prescribed dose of 12–35 Gy. Among these patients, six achieved a complete response, and six achieved a partial response, resulting in a 2-year overall survival (OS) rate of 47%. This study demonstrated that fractionated BNCT, administered at 30-day intervals with adaptive planning, is both effective and safe (122). Another study at the same center (NCT02004795) (135) focused on combining BNCT with image-guided intensity-modulated radiotherapy (IG-IMRT). The trial involved nine participants, and the combined BNCT+IMRT plan showed a significantly better conformity index for gross tumor volume (GTV) than did the BNCT-alone plan (P = 0.003). This improvement was particularly notable for tumors larger than 100 cm³, indicating that combining BNCT with IG-IMRT enhances homogeneity and conformity in treating larger tumors. This study aimed to enroll 28 patients, and the final survival-related results are anticipated.

A phase I/II trial (NCT00114790) from Finland involved 12 patients with locally advanced (rT3, rT4, or rN2) head and neck cancer that had recurred and was inoperable (124). This study revealed that 83% of patients responded positively to treatment, and 17% experienced tumor growth stabilization for 5.5 to 7.6 months. The median duration of response was 12.1 months, highlighting the effectiveness and favorable safety profile of BNCT for treating inoperable, locally advanced head and neck carcinomas, including those with recurrence at previously irradiated sites.

In Japan, BNCT research for HNC has also been substantial. Over the past 20 years, five trials have been registered. Hirose et al. conducted a trial (jRCT2080224571) using BNCT with a cyclotron-based epithermal neutron source (C-BENS). The study included 21 patients, with eight recurrent SCC (rSCC) and 13 non-SCC (r/la-nSCC) cases. The response rates were 42.9% at one month, 57.1% at two months, and 71.4% at three months (126). The two-year OS rates were 58% for the rSCC group and 100% for the non-SCC group. Additionally, two small-sample phase II studies initiated in 2019 have yet to publish any results. By 2023, two other trials (UMIN000044118 and ChiCTR2200066473) established by Japanese and Chinese researchers were registered for HNC and BNCT. The Japanese study is expected to include 120 patients with SCC of the head and neck, providing valuable insights from larger clinical research outcomes.

**Melanoma:** Boron–Neutron capture therapy (BNCT) has demonstrated efficacy in treating melanoma, particularly in advanced stages. Research suggests that BNCT is effective in reducing tumor size and controlling metastases. Since 2013, five studies have investigated BNCT for skin tumors, but only one study has published results. A phase I/II trial conducted by Kamitani (136) (jRCTs061180066) and completed in June 2020 included three patients with recurrent skin malignancies who had not undergone surgical treatment. The patients were intravenously administered the BPA-F complex (200 mg/kg body weight) for 2.5 to 3 hours before irradiation. The results revealed a 100% tumor control rate (CR+PR) with no adverse effects exceeding Grade 3. These findings indicate the effectiveness of BNCT against recurrent skin cancers and malignancies. However, the study included only three patients. The largest ongoing study is being conducted by researchers from Xiangya Third Hospital in China. This study aimed to enroll 30 patients, use 350 mg/kg BPA and deliver 20.0 Gy of radiation biological effectiveness (RBE). The results of this study are anticipated and may provide further insights into the efficacy of BNCT for the treatment of melanoma.

In addition to the tumors mentioned above, BNCT has also been investigated in other cancers, such as liver cancer, sarcoma, angiosarcoma, and refractory breast cancer, demonstrating objective efficacy. For example, a phase I/II clinical study published in 2015 (137) reported the treatment of malignant peripheral nerve sheath tumors (MPNSTs) with 500 mg/kg of boronophenylalanine (BPA) and 24.3 Gy-Eq, resulting in a two-year stable disease (SD) period. Other nonregistered studies are not discussed here.

### 4.4 Future research trends

Although BNCT was theoretically established more than 80 years ago, its clinical development has been limited by neutron source technology. Recent advancements in accelerator-driven neutron sources have revitalized BNCT, enabling practical applications. On the basis of the analysis of recent keyword clusters, future research will likely focus on several key areas: enhancing targeting and specificity through new boron carriers that selectively accumulate in tumors, improving molecular targeting strategies, and developing more accurate assays for boron uptake. Additionally, synergistic approaches that combine BNCT with other therapies, such as immunotherapy and advanced delivery systems using nanoparticles, will be explored to improve treatment efficacy. Establishing standardized radiation dose guidelines through clinical trials is essential for diverse cancer types, alongside efforts to increase international collaboration and innovation in neutron source development to increase the accessibility and cost effectiveness of BNCT.

### 4.5 Advantages and limitations

The primary strength of our research lies in the comprehensive analysis of global publications on BNCT from a scientific literature perspective. However, there are several limitations. First, the papers in this study were exclusively sourced from WOSCC, which may have resulted in some omissions in the literature, especially considering the long-time frame. Second, since our findings were primarily based on common bibliometric indicators, we may have overlooked important metrics, potentially missing valuable insights. Third, as the BNCT field is rapidly advancing, the most recent publications were not included in our analysis; these publications will be incorporated in future studies. Fourth, the retrieval strategy focused solely on publications written in English, potentially introducing selection bias by excluding studies in other languages. Finally, owing to the large sample size, our findings provide a general overview of the BNCT field, but some potentially valuable research directions may have been overlooked.

## 5 Conclusion

To summarize, we conducted a bibliometric analysis using VOSviewer, R, and CiteSpace to outline the current research situation and development trend of BNCT. This article demonstrates the characteristics of the publications; identifies the most influential countries, institutions, authors, journals, and articles; and analyzes the bursts of keywords and references. In addition, we also discussed the research hotspots and trends of BNCT. At present, basic and preclinical research on novel boron agents, as well as the clinical application of BNCT in brain tumors, head and neck malignancies and melanomas, are current research hotspots. Future research trends will include improving the treatment regimen, examining the application of accelerator-based neutron sources, refining the dosimetry, and exploring the fitness of BNCT in other clinical settings, such as multiple liver or lung metastases. In addition, the synergistic effects and cost effectiveness of BNCT are largely unstudied. Finally, the pursuit of a better boron delivery solution is still far from complete.

## Data Availability

The original contributions presented in the study are included in the article/**Supplementary Material**. Further inquiries can be directed to the corresponding authors.
