# Supplementary material for: Current research trends and hotspots of boron neutron capture therapy: a bibliometric and visualization analysis
Source: Front Oncol. 2024 Dec 12;14:1507157. doi: 10.3389/fonc.2024.1507157 (PMC11669655; doi:10.3389/fonc.2024.1507157)
Supplement: Supplementary file 1 [file Table1.docx]

Supplementary table 1. Most cited publications on BNCT each year from 1975 to 2023

| Year | Author | Title | Journal | Citations |
| --- | --- | --- | --- | --- |
| 1975 | HATANAKA H | A revised boron-neutron capture therapy for malignant brain tumors | J NEUROL | 80 |
| 1976 | NAKAGAWA T | Interaction between Serum Albumin and Mercaptoundecahydrododecaborate Ion (An Agent for Boron-Neutron Capture Therapy of Brain Tumor). I. Introductory Remarks and Preliminary Experiments | CHEM PHARM BULL | 19 |
| 1978 | IKEUCHI I | A colorimetric determination of boron in biological materials | CHEM PHARM BULL | 29 |
| 1980 | AIZAWA O | Remodeling and dosimetry on the neutron irradiation facility of the Musashi Institute of Technology Reactor for boron neutron capture therapy | NUCL TECHNOL | 21 |
| 1981 | WATTS KP | Melanin content of hamster tissues, human tissues, and various melanomas | CANCER RES | 58 |
| 1982 | KOBAYASHI T | Analytical calculation of boron-10 dosage in cell nucleus for neutron capture therapy | RADIAT RES | 139 |
| 1983 | SLATKIN DN | Whole-body irradiation of deuterated mice by the 10B (n, alpha) 7Li reaction. | P NATL ACAD SCI-BIOL | 11 |
| 1984 | GOLDENBERG DM | Neutron-capture therapy of human cancer: in vivo results on tumor localization of boron-10-labeled antibodies to carcinoembryonic antigen in the GW-39 tumor model system. | P NATL ACAD SCI-BIOL | 43 |
| 1985 | FAIRCHILD RG | Current status of ^10^B-neutron capture therapy: Enhancement of tumor dose via beam filtration and dose rate, and the effects of these parameters on minimum boron content: A theoretical evaluation | INT J RADIAT ONCOL | 181 |
| 1986 | FAIRCHILD RG | Microanalytical techniques for boron analysis using the 10B(n,α)7Li reaction | MED PHYS | 83 |
| 1987 | GABEL D | The Monte Carlo Simulation of the Biological Effect of the 10B(n,α)7Li Reaction in Cells and Tissue and Its Implication for Boron Neutron Capture Therapy | RADIAT RES | 188 |
| 1988 | OENBRINK G | Accumulation of porphyrins in cells: influence of hydrophobicity aggregation and protein binding | PHOTOCHEM PHOTOBIOL | 90 |
| 1989 | ALAM F | Boron neutron capture therapy: linkage of a boronated macromolecule to monoclonal antibodies directed against tumor-associated antigens | J MED CHEM | 96 |
| 1990 | BARTH RF | Boron neutron capture therapy for cancer | CANCER RES | 297 |
| 1991 | SLATKIN DN | A history of boron neutron capture therapy of brain tumours: postulation of a brain radiation dose tolerance limit | BRAIN | 156 |
| 1992 | WYZLIC IM | A general, convenient way to carborane-containing amino acids for boron neutron capture therapy | TETRAHEDRON LETT | 51 |
| 1993 | YAMAMOTO Y | Regio- and stereo-selective ring opening of epoxides with amide cuprate reagents | J CHEM SOC CHEM COMM | 68 |
| 1994 | GROZIAK MP | Boron Heterocycles Bearing a Peripheral Resemblance to Naturally-Occurring Purines: Design, Syntheses, Structures, and Properties | J AM CHEM SOC | 75 |
| 1995 | KARNBROCK W | Enantioselective synthesis of S-o-carboranylalanine via methylated bislactim ethers of 2,5-diketopiperazines | TETRAHEDRON | 38 |
| 1996 | SWENSON DH | Synthesis and Evaluation of a Boronated Nitroimidazole for Boron Neutron Capture Therapy | J MED CHEM | 23 |
| 1997 | NAKAMURA H | Synthesis of Carboranes Containing an Azulene Framework and in Vitro Evaluation as Boron Carriers | J MED CHEM | 38 |
| 1998 | TIETZE LF | Ortho-Carboranyl Glycosides of Glucose, Mannose, Maltose and Lactose for Cancer Treatment by Boron Neutron-Capture Therapy | CHEM-EUR J | 59 |
| 1999 | GIOVENZANA GB | Synthesis of carboranyl derivatives of alkynyl glycosides as potential BNCT agents | TETRAHEDRON | 76 |
| 2000 | SIVAEV IB | Synthesis of oxonium derivatives of the dodecahydro-closo-dodecaborate anion [B12H12]2−. Tetramethylene oxonium derivative of [B12H12]2− as a convenient precursor for the synthesis of functional compounds for boron neutron capture therapy | POLYHEDRON | 104 |
| 2001 | KABALKA GW | Synthesis of novel boron containing unnatural cyclic amino acids as potential therapeutic agents | TETRAHEDRON LETT | 45 |
| 2002 | VALLIANT JF | Synthesis of Boroxifen, A Nido-Carborane Analogue of Tamoxifen | J ORG CHEM | 67 |
| 2003 | YANG WQ | Boronic acid compounds as potential pharmaceutical agents | MED RES REV | 439 |
| 2004 | OYEWUMI MO | Comparison of cell uptake, biodistribution and tumor retention of folate-coated and PEG-coated gadolinium nanoparticles in tumor-bearing mice | J CONTROL RELEASE | 254 |
| 2005 | GILLIES ER | Dendrimers and dendritic polymers in drug delivery | DRUG DISCOV TODAY | 1158 |
| 2006 | CODERRE JA | Late effects of radiation on the central nervous system: role of vascular endothelial damage and glial stem cell survival | RADIAT RES | 121 |
| 2007 | KANKAANRANTA L | Boron neutron capture therapy in the treatment of locally recurred head and neck cancer | INT J RADIAT ONCOL | 113 |
| 2008 | SEMIOSHKIN AA | Cyclic oxonium derivatives of polyhedral boron hydrides and their synthetic applications | DALTON T | 184 |
| 2009 | SIVAEV IB | Polyhedral Boranes for Medical Applications: Current Status and Perspectives | EUR J INORG CHEM | 316 |
| 2010 | BALDOCK C | Polymer gel dosimetry | PHYS MED BIOL | 707 |
| 2011 | SCHOLZ M | Carbaboranes as pharmacophores: properties, synthesis, and application strategies | CHEM REV | 629 |
| 2012 | BARTH RF | Current status of boron neutron capture therapy of high-grade gliomas and recurrent head and neck cancer | RADIAT ONCOL | 387 |
| 2013 | DAS BC | Boron chemicals in diagnosis and therapeutics | FUTURE MED CHEM | 195 |
| 2014 | MOSS RL | Critical review, with an optimistic outlook, on Boron Neutron Capture Therapy (BNCT) | APPL RADIAT ISOTOPES | 206 |
| 2015 | ASSAF KI | Water structure recovery in chaotropic anion recognition: high‐affinity binding of dodecaborate clusters to γ‐cyclodextrin | ANGEW CHEM INT EDIT | 211 |
| 2016 | NÚÑEZ R | Electrochemistry and photoluminescence of icosahedral carboranes, boranes, metallacarboranes, and their derivatives | CHEM REV | 396 |
| 2017 | LI X | Hollow boron nitride nanospheres as boron reservoir for prostate cancer treatment | NAT COMMUN | 141 |
| 2018 | BARTH RF | Boron delivery agents for neutron capture therapy of cancer | CANCER COMMUN | 296 |
| 2019 | DUKENBAYEV K | Fe3O4 Nanoparticles for Complex Targeted Delivery and Boron Neutron Capture Therapy | NANOMATERIALS-BASEL | 129 |
| 2020 | SUZUKI M | Boron neutron capture therapy (BNCT): A unique role in radiotherapy with a view to entering the accelerator-based BNCT era | INT J CLIN ONCOL | 159 |
| 2021 | MALOUFF TD | Boron neutron capture therapy: a review of clinical applications | FRONT ONCOL | 129 |
| 2022 | KANAI Y | Amino acid transporter LAT1 (SLC7A5) as a molecular target for cancer diagnosis and therapeutics | PHARMACOL THERAPEUT | 85 |
| 2023 | COGHI P | Next generation of boron neutron capture therapy (BNCT) agents for cancer treatment | MED RES REV | 27 |

Supplementary table 2. The top 100 most cited articles.

| Rank | Publication year | Authors | DOI | Journals | Total Citations |
| --- | --- | --- | --- | --- | --- |
| 1 | 2005 | GILLIES ER | 10.1016/S1359-6446(04)03276-3 | DRUG DISCOV TODAY | 1158 |
| 2 | 1993 | HAWTHORNE MF | 10.1002/anie.199309501 | ANGEW CHEM INT EDIT | 851 |
| 3 | 2005 | BARTH RF | 10.1158/1078-0432.CCR-05-0035 | CLIN CANCER RES | 849 |
| 4 | 2010 | BALDOCK C | 10.1088/0031-9155/55/5/R01 | PHYS MED BIOL | 707 |
| 5 | 2011 | SCHOLZ M | 10.1021/cr200038x | CHEM REV | 630 |
| 6 | 2002 | VALLIANT JF | 10.1016/S0010-8545(02)00087-5 | COORDIN CHEM REV | 612 |
| 7 | 1992 | PLESEK J | 10.1021/cr00010a005 | CHEM REV | 598 |
| 8 | 2011 | ISSA F | 10.1021/cr2000866 | CHEM REV | 578 |
| 9 | 2011 | MARUYAMA K | 10.1016/j.addr.2010.09.003 | ADV DRUG DELIVER REV | 495 |
| 10 | 1999 | HAWTHORNE MF | 10.1021/cr980442h | CHEM REV | 479 |
| 11 | 2003 | YANG WQ | 10.1002/med.10043 | MED RES REV | 443 |
| 12 | 1999 | CODERRE JA | 10.2307/3579742 | RADIAT RES | 434 |
| 13 | 2016 | NÚÑEZ R | 10.1021/acs.chemrev.6b00198 | CHEM REV | 400 |
| 14 | 2012 | BARTH RF | 10.1186/1748-717X-7-146 | RADIAT ONCOL | 387 |
| 15 | 2000 | RAPOPORT SI | 10.1023/A:1007049806660 | CELL MOL NEUROBIOL | 352 |
| 16 | 2011 | CAMBRE JN | 10.1016/j.polymer.2011.07.057 | POLYMER | 348 |
| 17 | 2009 | SIVAEV IB | 10.1002/ejic.200900003 | EUR J INORG CHEM | 316 |
| 18 | 2018 | BARTH RF | 10.1186/s40880-018-0299-7 | CANCER COMMUN | 299 |
| 19 | 1990 | BARTH RF | NA | CANCER RES | 297 |
| 20 | 1992 | BARTH RF | NA | CANCER-AM CANCER SOC | 295 |
| 21 | 2003 | OTSUKA H | 10.1021/ja021303r | J AM CHEM SOC | 287 |
| 22 | 2009 | BAKER SJ | 10.4155/FMC.09.71 | FUTURE MED CHEM | 267 |
| 23 | 1994 | BARTH RF | 10.1021/bc00025a008 | BIOCONJUGATE CHEM | 260 |
| 24 | 2004 | OYEWUMI MO | 10.1016/j.jconrel.2004.01.002 | J CONTROL RELEASE | 254 |
| 25 | 2016 | LESNIKOWSKI ZJ | 10.1021/acs.jmedchem.5b01932 | J MED CHEM | 252 |
| 26 | 2004 | KATO I | 10.1016/j.apradiso.2004.05.059 | APPL RADIAT ISOTOPES | 229 |
| 27 | 1999 | KUSHNER DJ | 10.1139/cjpp-77-2-79 | CAN J PHYSIOL PHARM | 228 |
| 28 | 1994 | HATANAKA H | 10.1016/0360-3016(94)90479-0 | INT J RADIAT ONCOL | 220 |
| 29 | 2015 | ASSAF KI | 10.1002/anie.201412485 | ANGEW CHEM INT EDIT | 215 |
| 30 | 2014 | MOSS RL | 10.1016/j.apradiso.2013.11.109 | APPL RADIAT ISOTOPES | 207 |
| 31 | 2004 | GRIMES RN | NA | J CHEM EDUC | 203 |
| 32 | 2005 | YINGHUAI Z | 10.1021/ja0517116 | J AM CHEM SOC | 198 |
| 33 | 2013 | DAS BC | 10.4155/FMC.13.38 | FUTURE MED CHEM | 197 |
| 34 | 1997 | SAH RN | 10.1006/mchj.1997.1428 | MICROCHEM J | 196 |
| 35 | 1987 | GABEL D | 10.2307/3577018 | RADIAT RES | 188 |
| 36 | 2008 | SEMIOSHKIN AA | 10.1039/b715363e | DALTON T | 184 |
| 37 | 2006 | PERRY A | 10.1007/s00401-005-0023-y | ACTA NEUROPATHOL | 182 |
| 38 | 1985 | FAIRCHILD RG | 10.1016/0360-3016(85)90318-9 | INT J RADIAT ONCOL | 181 |
| 39 | 2001 | ENDO Y | 10.1016/S1074-5521(01)00016-3 | CHEM BIOL | 180 |
| 40 | 2013 | CIOFANI G | 10.1002/smll.201201315 | SMALL | 178 |
| 41 | 1999 | BARTH RF | 10.1097/00006123-199903000-00001 | NEUROSURGERY | 174 |
| 42 | 1999 | CHANANA AD | 10.1097/00006123-199906000-00013 | NEUROSURGERY | 173 |
| 43 | 1998 | DEMARCO JJ | 10.1118/1.598167 | MED PHYS | 169 |
| 44 | 2003 | HAWTHORNE MF | 10.1007/BF02699932 | J NEURO-ONCOL | 168 |
| 45 | 2004 | MARUYAMA K | 10.1016/j.jconrel.2004.04.018 | J CONTROL RELEASE | 167 |
| 46 | 2020 | SUZUKI M | 10.1007/s10147-019-01480-4 | INT J CLIN ONCOL | 163 |
| 47 | 2004 | WU G | 10.1021/bc0341674 | BIOCONJUGATE CHEM | 161 |
| 48 | 2003 | JOENSUU H | 10.1023/A:1023293006617 | J NEURO-ONCOL | 160 |
| 49 | 2010 | SIVAEV IB | 10.1135/cccc2010054 | COLLECT CZECH CHEM C | 159 |
| 50 | 2018 | BARTH RF | 10.1186/s40880-018-0280-5 | CANCER COMMUN-a | 159 |
| 51 | 2020 | ALI F | 10.3390/molecules25040828 | MOLECULES | 157 |
| 52 | 1991 | SLATKIN DN | 10.1093/brain/114.4.1609 | BRAIN | 156 |
| 53 | 2005 | BACKER MV | 10.1158/1535-7163.MCT-05-0161 | MOL CANCER THER | 153 |
| 54 | 1992 | SHELLY K | 10.1073/pnas.89.19.9039 | P NATL ACAD SCI USA | 150 |
| 55 | 2020 | HOPPENZ P | 10.3389/fchem.2020.00571 | FRONT CHEM | 150 |
| 56 | 1997 | NAKAGAWA Y | 10.1023/A:1005781517624 | J NEURO-ONCOL | 149 |
| 57 | 2003 | SHUKLA S | 10.1021/bc025586o | BIOCONJUGATE CHEM | 149 |
| 58 | 2015 | KALAY S | 10.3762/bjnano.6.9 | BEILSTEIN J NANOTECH | 146 |
| 59 | 1993 | CODERRE JA | 10.1016/0360-3016(93)90533-2 | INT J RADIAT ONCOL | 145 |
| 60 | 1998 | IMAHORI Y | NA | J NUCL MED | 145 |
| 61 | 2009 | CIOFANI G | 10.1007/s11671-008-9210-9 | NANOSCALE RES LETT | 145 |
| 62 | 1994 | MORIN C | 10.1016/S0040-4020(01)89389-3 | TETRAHEDRON | 144 |
| 63 | 1998 | HAWTHORNE MF | 10.1016/S1357-4310(98)01226-X | MOL MED TODAY | 144 |
| 64 | 1998 | CODERRE JA | 10.2307/3579926 | RADIAT RES | 144 |
| 65 | 1998 | TSUBOI T | 10.1111/j.1600-0749.1998.tb00736.x | PIGM CELL RES | 142 |
| 66 | 2017 | LI X | 10.1038/ncomms13936 | NAT COMMUN | 142 |
| 67 | 2020 | DYMOVA MA | 10.1002/cac2.12089 | CANCER COMMUN | 141 |
| 68 | 1992 | HILL JS | 10.1073/pnas.89.5.1785 | P NATL ACAD SCI USA | 140 |
| 69 | 2005 | PARROTT MC | 10.1021/ja053730l | J AM CHEM SOC | 140 |
| 70 | 1982 | KOBAYASHI T | 10.2307/3575817 | RADIAT RES | 139 |
| 71 | 2011 | QIN ZY | 10.1039/c0mt00048e | METALLOMICS | 135 |
| 72 | 1999 | LEE CL | 10.1016/S0168-583X(99)00026-9 | NUCL INSTRUM METH B | 134 |
| 73 | 2016 | SINGH B | 10.1038/srep35535 | SCI REP-UK | 133 |
| 74 | 1994 | NEWKOME GR | 10.1002/anie.199406661 | ANGEW CHEM INT EDIT | 132 |
| 75 | 1997 | BARTH RF | NA | CANCER RES | 132 |
| 76 | 2020 | HU K | 10.1016/j.ccr.2019.213139 | COORDIN CHEM REV | 132 |
| 77 | 2003 | CAPALA J | 10.1007/BF02699940 | J NEURO-ONCOL | 131 |
| 78 | 2021 | MALOUFF TD | 10.3389/fonc.2021.601820 | FRONT ONCOL | 131 |
| 79 | 2021 | ZHAO X | 10.1016/j.ccr.2021.214042 | COORDIN CHEM REV | 131 |
| 80 | 1994 | CODERRE JA | 10.1016/0360-3016(92)90951-D | INT J RADIAT ONCOL | 130 |
| 81 | 1997 | CODERRE JA | 10.1023/A:1005741919442 | J NEURO-ONCOL | 129 |
| 82 | 2003 | NAKAGAWA Y | 10.1007/BF02699936 | J NEURO-ONCOL | 129 |
| 83 | 2019 | DUKENBAYEV K | 10.3390/nano9040494 | NANOMATERIALS-BASEL | 129 |
| 84 | 2000 | WITTIG A | 10.1667/0033-7587(2000)153[0173:MOTOPB]2.0.CO;2 | RADIAT RES | 128 |
| 85 | 2016 | LYU HR | 10.1021/jacs.6b07086 | J AM CHEM SOC | 125 |
| 86 | 2019 | TISHKEVICH DI | 10.1016/j.jallcom.2019.05.075 | J ALLOY COMPD | 125 |
| 87 | 2014 | SUZUKI M | 10.1093/jrr/rrt098 | J RADIAT RES | 124 |
| 88 | 2015 | BAN HS | 10.1002/tcr.201402100 | CHEM REC | 124 |
| 89 | 2016 | HANSEN BRS | 10.1016/j.ccr.2015.12.003 | COORDIN CHEM REV | 124 |
| 90 | 2017 | NAGARAJAN S | 10.1021/acsami.7b13199 | ACS APPL MATER INTER | 123 |
| 91 | 2006 | CODERRE JA | 10.1667/RR3597.1 | RADIAT RES | 121 |
| 92 | 1996 | CAPALA J | 10.1021/bc950077q | BIOCONJUGATE CHEM | 118 |
| 93 | 2008 | YAMAMOTO T | 10.1016/j.canlet.2008.01.021 | CANCER LETT | 118 |
| 94 | 2021 | OU MT | 10.1002/advs.202001801 | ADV SCI | 118 |
| 95 | 2011 | NONOGUCHI N | 10.1007/s11060-011-0610-9 | J NEURO-ONCOL | 117 |
| 96 | 2011 | TOUCHET S | 10.1039/c0cs00154f | CHEM SOC REV | 117 |
| 97 | 1994 | FEAKES DA | 10.1073/pnas.91.8.3029 | P NATL ACAD SCI USA | 115 |
| 98 | 2003 | BLUE TE | NA | J NEURO-ONCOL | 115 |
| 99 | 2017 | CHENG RF | 10.1038/ncomms14827 | NAT COMMUN | 115 |
| 100 | 2007 | KANKAANRANTA L | 10.1016/j.ijrobp.2007.03.039 | INT J RADIAT ONCOL | 114 |

Supplementary table 3. Eight clusters of keywords regarding BNCT research in last 5 years.

| No. | Cluster center | Size | Silhouette value |
| --- | --- | --- | --- |
| 0 | BPA | 58 | 0.635 |
| 1 | Boron | 44 | 0.737 |
| 2 | Epithermal neutron | 44 | 0.702 |
| 3 | Drug delivery | 34 | 0.783 |
| 4 | Beam | 33 | 0.753 |
| 5 | Derivatives | 27 | 0.770 |
| 6 | Growth | 23 | 0.809 |
| 7 | Biodistrbution | 17 | 0.822 |
